# Oxidative Modifications of Rat Liver Cell Components During *Fasciola hepatica* Infection

**DOI:** 10.1080/15376510701624001

**Published:** 2008-06-23

**Authors:** Ewa Siemieniuk, Lidia Kolodziejczyk, Elżbieta Skrzydlewska

**Affiliations:** Department of Inorganic and Analytical Chemistry, Medical University of Bialystok, Poland; Department of Biology and Medical Parasitology, Pomeranian Medical University, Szczecin, Poland; Department of Inorganic and Analytical Chemistry, Medical University of Bialystok, Poland

**Keywords:** Fasciola hepatica, Fasciolosis, Lipid Peroxidation, Oxidative Modifications

## Abstract

The aim of this paper was to assess the influence of *Fasciola hepatica* infection on oxidative modifications of rat liver cell components such as proteins and lipids. Wistar rats were infected per os with 30 metacercariae of *F. hepatica*. Activities and concentrations of liver damage markers were determined in the 4th, 7th, and 10th week postinfection (wpi). A decrease in antioxidant capacity of the host liver, manifested by a decrease in total antioxidant status (TAS), was observed. Diminution of antioxidant abilities resulted in enhanced oxidative modifications of lipids and proteins. *F. hepatica* infection enhanced lipid peroxidation, which was visible in the statistically significant increase in the level of different lipid peroxidation products such as conjugated dienes (CDs), lipid hydroperoxides (LOOHs), malondialdehyde (MDA) and 4-hydroxynonenal (4-HNE). The level of protein modification markers in the rat liver was also significantly changed and the most intensified changes were observed at seventh week postinfection. Concentration of carbonyl groups and dityrosine was significantly increased, whereas the level of tryptophan and sulfhydryl and amino groups was decreased. Changes in the antioxidant abilities of the liver and in the lipid and protein structure of the cell components resulted in destruction of the function of the liver. *F. hepatica* infection was accompanied by raising serum activities of alanine aminotransferase (ALT) and aspartate aminotransferase (AST) as markers of liver damage. A significant decrease in lysosomal as well as in the total activity of cathepsin B during fasciolosis was also observed.

## INTRODUCTION

*Fasciola hepatica* is the cosmopolitan fluke parasitizing in the liver parenchyma and bile ducts, mostly in herbivorous wild and domestic animals (sheep, goats, cattle) as well as in humans. Due to an increase in the number of cases of this fluke infection in humans all over the world, fasciolosis has been classified by the World Health Organization (WHO) as a serious threat to public health (Mas-Coma et al. 1999, 2005). Currently, according to various assessments, 2.4 million (WHO 1995) to as many as 17 million (Hopkins 1992) of the world population may be infected by *F. hepatica*, and the risk of *F. hepatica* infection concerns over 180 million of the population (WHO 1995). The greatest number of clinical cases of human fasciolosis was recognized in Bolivia, Ecuador, Egypt, France, Iran, Peru, and Portugal (WHO 1995).

Hepatic lesions produced by *F. hepatica* are invariably associated with chemical alterations in the cell, such as enhancement of membrane lipid peroxidation and marked suppression of the microsomal drug-metabolizing mono-oxygenase system (Maffei Facino et al. 1989, 1993; Abdel Rehim et al. 2003). Uncoupling of oxidative phosphorylation, inhibition of respiratory reactions, and loss of oligomycin sensitivity by the F_1_F_0_-ATPase were demonstrated in mitochondria of rat hepatocytes (Rule et al. 1989; Hanisch et al. 1992; Lenton et al. 1994). In rat fasciolosis, hepatocyte mitochondria accumulate free fatty acids and are also depleted of phospholipids resulting in loss of membrane integrity (Lenton et al. 1995) and other structural and functional changes.

It is generally believed that the host is capable of significant tolerance to infection by the liver fluke thanks to defense mechanisms associated with the generation of reactive oxygen species (ROS) (Smith et al. 1992; Maffei Facino et al. 1993; Abu-Shousha et al. 1999).

In the course of fasciolosis, enhanced generation of reactive oxygen species (ROS) and reactive nitrogen species (RNS) is observed. ROS generation by peritoneal leucocytes in response to the *Fasciola* antigen was found particularly in the course of secondary invasion in rats (Smith et al. 1992). An increase in superoxide radical anion (O_2_^−.^) as well as in the NO level, generated through monocytes, was revealed during chronic fasciolosis in humans (Abo-Shousha et al. 1999) and also through peritoneal cells in rats (Piedrafita et al. 2001; Sibille et al. 2004).

Independently of increased free radical generation *F. hepatica* infection is accompanied by disturbances in antioxidant mechanisms, which lead to ineffectiveness in ROS scavenging (Kolodziejczyk et al. 2005). In such a situation ROS are helpful in host tolerance to infection but also the high chemical reactivity of ROS may lead to reactions with almost all constituents of the cell, including proteins, lipids, and DNA. Attack by ROS causes the alterations in molecular structure and biological properties (Berlett and Stadtman 1997; Peskin 1997; Fujita 2002), which may be detrimental to the cell of host liver. Therefore, we have decided to examine the effects of *F. hepatica* infection on rat liver antioxidant abilities and in consequence on lipids' and proteins' oxidative modifications.

## MATERIAL AND METHODS

### Animal Treatment

The experiment was done on male Wistar rats aged 5 weeks. The rats were housed in groups with free access to granulated standard food and water and a normal light-dark cycle was maintained. The study protocol was approved by the Local Bioethics Committee in Szczecin (Poland) in accordance with the Polish Animals Protection Act of 1997. Rats were infected per os with 30 metacercariae of *F. hepatica* administered through a stomach tube. Metacercariae were obtained from *Lymnaea truncatula* snail culture according to Taylor and Mozley (1948) and were classified as viable only if excretory granules were seen under an optical microscope (Boray 1969). Livers were removed in 10 control and 10 *F. hepatica* infected rats that had been anesthetized with ketamine at the 4th, 7th, and 10th week postinfection (wpi).

### Preparation of Tissue

Livers were placed in ice-cold 0.15 M NaCl solution, perfused with the same solution to remove blood cells, blotted on filter paper, weighed, and homogenized in 9 mL ice-cold 0.25 M sucrose and 0.15 M NaCl with the addition of 6 μL 250 mM BHT (butylated hydroxytoluene) in ethanol to prevent the formation of new peroxides during the assay. The homogenization procedure was performed under standardized conditions; 10% homogenates were centrifuged at 10,000 × g for 15 min at 4°C and the supernatant was kept on ice until assayed.

To assay protein oxidation, liver samples were homogenized in 5 mM phosphate buffer (pH 7.5) with protease inhibitors (leupeptin 0.5 mg/mL, aprotinin 0.5 mg/mL, pepstatin 0.7 mg/mL) and 0.1% Triton X-100. The homogenate was centrifuged at 7800 × g for 20 min and the biochemical analysis were performed on the supernatant.

To assay cathepsin B activity liver samples were homogenized in a glass Teflon Potter homogenizer in 0.25 M sucrose without and with 0.2% Triton X-100. The homogenates were centrifuged at 100,000 × g (4°C) for 60 min to settle the organelles or their membranes. Supernatant received from homogenate prepared in sucrose was called cytosol. Supernatant received from homogenate prepared in sucrose with Triton X-100 was called the homogenate.

### Biochemical Assays

Lipid peroxidation was assayed by HPLC measurement of lipid hydroperoxides (LOOH) (Tokumaru et al. 1995), malondialdehyde (MDA) as a malondialdehyde-thiobarbituric acid adduct (Londero and Greco 1996), and 4-hydroxynonenal (4-HNE) as a fluorimetric derivative (Yoshino et al. 1986), and by spectrophotometrical measurement of conjugated dienes (CD) at 234 nm (Recknogel and Glende 1984).

Protein oxidative modifications were examined by determination of carbonyl group, dityrosine, tryptophan, sulfhydryl group, and amino group levels. Carbonyl groups were determined spectrophotometrically using 2,4-dinitrophenylhydrazine (Levine et al. 1990). Dityrosine content was estimated by fluorescence spectrophotometry at 325 nm excitation and 420 nm emission (Rice-Evans at al. 1991). Fluorescence emission at 338 nm and excitation at 288 nm was used as a reflection of tryptophan content (Rice-Evans at al. 1991). The free amino groups were assayed by reaction with ninhydrin (Devenyi and Gregely 1968), while sulphydryl groups were determined by the Ellman reaction (Ellman 1959).

In the liver cytosol and homogenate the activity of cathepsin B was determined with Bz-DL-Arg-pNA (Sigma Chemical Co, St. Louis, USA) as a substrate, at pH 6.0, by measuring released p-nitroaniline at 405 nm during 2 h incubation at 37°C (Tawatari et al. 1979). The activity of cathepsin B in lysosomes was calculated as a difference in activity in homogenate and in cytosol. The protein concentration was determined according to Lowry et al. (1951).

Diagnostic Biomerieux tests were used for assessment of blood serum alanine aminotransferase (ALT) and aspartate aminotransferase (AST) activities.

### Statistical Analysis

The data obtained in this study are expressed as mean ± SD. The data were analyzed by use of standard statistical analyses, one-way ANOVA, with Scheffe's F-test for multiple comparisons to determine significance between different groups. The values for *p* < 0.05 were considered significant.

## RESULTS

*F. hepatica* infection caused a significant decrease in antioxidant capacity of the host liver, which was manifested by a significant decrease in total antioxidant status (TAS) by about 15%, 16%, and 12% in comparison with control group in the 4th, 7th, and 10th wpi, respectively (Table 1).

**TABLE 1:** TAS in the liver of control and *F. hepatica*-infected rats at 4, 7, and 10 wpi

	Weeks postinfection					
	
	4		7		10	
			
	Control rats	Infected rats	Control rats	Infected rats	Control rats	Infected rats
TAS mol/g tissue	110.6 ± 6.5	93.5 ± 7.3^a^	108.2 ± 6.9	91.4 ± 7.5^a^	112.5 ± 6.1	98.8 ± 7.7^a^

a Significantly different from control group (*p* <0.05).

Diminution of antioxidant abilities resulted in enhanced oxidative modifications of liver cell components such as lipids and proteins. Infection with *F. hepatica* enhanced lipid peroxidation, which was visible in the significant increase in the level of different lipid peroxidation products. The level of the first lipid peroxidation product—conjugated dienes (CDs)—has a tendency to increase (by about 5% and 15% in comparison with control groups in the 4th and 10th wpi, respectively). However, the lipid hydroperoxide (LOOH) level was increased (by about 51%, 48%, and 31% in comparison with control groups in the 4th, 7th, and 10th wpi, respectively). The content of malondialdehyde (MDA) and 4-hyroxynonenal (4-HNE) was significantly increased by about 97%, 75%, and 69% for MDA and by about 108%, 99%, and 73% for 4-HNE in comparison with control groups in the 4th, 7th, and 10th wpi, respectively (Table 2).

**TABLE 2:** The levels of lipid peroxidation products: conjugated dienes (CDs) lipid hydroperoxides (LOOHs), malondialdehyde (MDA), and 4-hydroxynonenal (4-HNE) in the liver of control and *F. hepatica*-infected rats at 4, 7, and 10 wpi

	Weeks postinfection					
	
	4		7		10	
			
	Control rats	Infected rats	Control rats	Infected rats	Control rats	Infected rats
CD (μmol/g tissue)	1.27 ± 0.05	1.34 ± 0.12	1.30 ± 0.06	1.39 ± 0.14	1.29 ± 0.06	1.48 ± 0.14
LOOH (μmol/g tissue)	124 ± 8	187 ± 14	122 ± 8	181 ± 15	127 ± 9	167 ± 14
MDA (nmol/g tissue)	2.58 ± 0.15	5.09 ± 0.52^a^	2.74 ± 0.15	4.79 ± 0.52^a^	2.71 ± 0.15	4.58 ± 0.52^a^
4-HNE (nmol/g tissue)	1.36 ± 0.08	2.84 ± 0.29^a^	1.27 ± 0.08	2.53 ± 0.23^a^	1.32 ± 0.23^a^	2.28 ± 0.23^a^

a Significantly different from control group (*p* < 0.05).

Table 3 shows the level of protein modification markers in the rat liver. The levels of carbonyl groups and dityrosine were significantly increased during fasciolosis by about 21%, 34%, and 38% for carbonyl groups and by about 28%, 68%, and 30% for dityrosine in comparison with control groups in the 4th, 7th, and 10th wpi, respectively. However, the level of tryptophan was statistically decreased by about 10% in comparison with control group only in the 7th wpi. The levels of sulfhydryl and amino groups were significantly decreased during fasciolosis by about 13%, 20%, and 15% for sulfhydryl groups and by about 11%, 21%, and 15% for amino groups in comparison with control group in the 4th, 7th, and 10th wpi, respectively.

**TABLE 3:** The levels of protein modification markers: carbonyl groups, dityrosine, tryptophan, sulfhydryl groups, and amino groups in the liver of control and *F. hepatica*-infected rats at 4, 7, and 10 wpi

	Weeks postinfection					
	
	4		7		10	
			
	Control rats	Infected rats	Control rats	Infected rats	Control rats	Infected rats
Carbonyl groups (nmol/mg protein)	0.97 ± 0.05	1.17 ± 0.08^a^	0.99 ± 0.06	1.32 ± 0.09^a^	0.97 ± 0.06	1.35 ± 0.11^a^
Dityrosine (U/mg protein)	0.36 ± 0.02	0.46 ± 0.04^a^	0.34 ± 0.02	0.57 ± 0.05^a^	0.40 ± 0.03	0.52 ± 0.04^a^
Tryptophan (U/mg protein)	7.91 ± 0.45	7.43 ± 0.58	7.95 ± 0.51	7.19 ± 0.63^a^	8.01 ± 0.55	7.42 ± 0.67
Sulfhydryl groups (nmol/mg protein)	4.11 ± 0.25	3.59 ± 0.31^a^	4.07 ± 0.27	3.24 ± 0.30^a^	4.09 ± 0.27	3.47 ± 0.30^a^
Amino groups (nmol Tyr/mg protein)	47.2 ± 3.0	42.1 ± 3.1^a^	47.6 ± 2.9	37.5 ± 3.1^a^	46.1 ± 3.1	39.1 ± 3.3^a^

a Significantly different from control group (*p* < 0.05).

A significant decrease in lysosomal as well as in the total activity of cathepsin B during fasciolosis was also observed (by about 18%, 23%, and 15% for lysosomal and by about 40%, 42%, and 25% for total in comparison with control group in the 4th, 7th, and 10th wpi, respectively). In consequence, the statistical increase by about 45%, 113%, and 68% in cytosol activity of cathepsin B was observed in comparison with control group in the 4th, 7th, and 10th wpi, respectively (Table 4).

**TABLE 4:** Activity of cathepsin B (pNA, μmol/g tissue) in the liver homogenate, cytosol, and lysosomes of control and *F. hepatica*-infected rats at 4, 7, and 10 wpi

	Weeks postinfection					
	
	4		7		10	
			
Localization	Control rats	Infected rats	Control rats	Infected rats	Control rats	Infected rats
Homogenate	1.87 ± 0.12	1.54 ± 0.13^a^	1.84 ± 0.11	1.42 ± 0.12^a^	1.89 ± 0.12	1.61 ± 0.14^a^
Cytoso	l0.22 ± 0.02	0.32 ± 0.03^a^	0.22 ± 0.02	0.47 ± 0.04^a^	0.22 ± 0.02	0.37 ± 0.03^a^
Lysosomes	1.65 ± 0.09	0.99 ± 0.08^a^	1.65 ± 0.09	0.95 ± 0.08^a^	1.65 ± 0.09	1.24 ± 0.10^a^

a Significantly different from control group (*p* < 0.05).

Damage of liver cells as a result of oxidative modification of liver cell components was also visible in the serum activities of the liver damage markers (ALT and AST). Table 5 shows that serum activities of alanine aminotransferase and aspartate aminotransferase were significantly increased during infection with *F. hepatica*. Almost a threefold increase in ALT and AST activities was observed in the infected group of rats in comparison with control group (by about 187%, 178%, and 194% for ALT and by about 120%, 116%, and 118% for AST in the 4th, 7th, and 10th wpi, respectively).

**TABLE 5:** Alanine aminotransferase (ALT) and aspartate aminotransferase (AST) activities in the serum of control and *F. hepatica*-infected rats at 4, 7, and 10 wpi

	Weeks postinfection					
	
	4		7		10	
			
	Control rats	Infected rats	Control rats	Infected rats	Control rats	Infected rats
ALT (U/L)	32.6 ± 1.4	93.7 ± 6.9^a^	34.5 ± 1.4	96.1 ± 6.9^a^	33.4 ± 1.6	98.3 ± 4.7^a^
AST (U/L)	162.1 ± 6.5	357.1 ± 26.7^a^	167.3 ± 6.5	361.8 ± 26.7^a^	164.2 ± 6.9	359.3 ± 24.6^a^

a Significantly different from control group (*p* < 0.05).

## DISCUSSION

It has been reported that ROS generation is enhanced during *F. hepatica* infection (Piedrafita et al. 2001). This fact is very important regarding the significant decrease in antioxidant capacity of the host liver after invasion, which was manifested by the decrease in activity/level of basic cellular enzymatic and nonenzymatic antioxidants shown in previous studies (Callahan et al. 1988; Kolodziejczyk et al. 2006) as well as a decrease in total antioxidant status observed in this study. Disturbances in oxidant-antioxidant balance existing in the organism may result in the higher exposure of cell components to the ROS action. It is known that proteins are major targets for ROS, which are formed in vivo both intra- and extracellularly. On the basis of rate constants and the knowledge of the relative abundance of macromolecules within cells, it has been estimated that proteins can scavenge 50% to 75% of ROS such as hydroxyl radical generated within a cell by γ-radiolysis (Gebicki and Gebicki 1993). All proteins are susceptible to attack by ROS, but some of them are more vulnerable than others (Stadtman and Levine 2003). Moreover, from amino acids composing protein moiety the most sensitive to oxidation are side chains of aromatic amino acids: tryptophan, tyrosine, and phenylalanine. Our study has proved that during *F. hepatica* infection the amount of tryptophan residues was significantly decreased, whereas the level of dityrosine, the product of ROS reaction with tyrosine, was increased in comparison to control. Dityrosine production appears to be a useful “marker” for protein modification especially by hydroxyl radical (Davies et al. 1999; Gebicki 2006). Another amino acid that is extremely sensitive to ROS action is cysteine. Most reports show that cysteine-the-cystine ratio in the proteins subjected to oxidation is decreased (Thannhauser et al. 1998). Several other amino acid residues in proteins such as prolyl, lysyl, and methionyl are modified by ROS, which result in changes in protein structure (Croft et al. 2003). ROS attack on lysine residue is manifested by a decrease in free amino groups. Our investigations showed the diminution in the level of protein sulphydryl and amino groups after *F. hepatica* infection. Moreover, disruption of polypeptide chain may also occur under certain conditions during oxidative stress when generated ROS act on the α-carbon atom of the peptide bond (Davies et al. 1999). ROS attack causes the fragmentation of polypeptide chain and formation of new carbonyl groups (Davies et al. 1999). In the present study the increase in the amount of protein carbonyl groups during *F. hepatica* infection has also been noticed. Modifications of individual amino acid residues may result in changes in secondary and tertiary structure of proteins and in consequence in their functions.

Independently of protein modifications caused directly by ROS, protein structure can also be changed by reactions with lipid oxidative modification products generated during *F. hepatica* infection, because the decrease in antioxidant abilities led also to intensification of lipid peroxidation observed in this paper. ROS reacting with unsaturated fatty acids, which mainly form membrane phospholipids, produce as first conjugated dienes and lipid hydroperoxides. It has been also shown in this paper that levels of conjugated dienes and lipid peroxides were enhanced during *F. hepatica* infection. It was reported that the transition metal ions break down lipid hydroperoxides to secondary lipid peroxidation products. A variety of compounds, which could be produced from such decomposition, may exert secondary toxic effects on cells, but the most reactive are malondialdehyde and 4-hydroxynonenal, as well as other carbonyls (Aust et al. 1985; Esterbauer et al. 1991). Observed in our research, the increase in the amount of final lipid peroxidation products (MDA and 4-HNE) indicates that the reactions of ROS with polyunsaturated fatty acids of phospholipid membranes occur intensively during *F. hepatica* infection. These carbonyl compounds are highly reactive and may act as “secondary toxic messengers” of the primary ROS event. The high reactivity of 4-HNE can be attributed to its α,β-unsaturated configuration, which gives to this biogenic aldehyde strong electrophilic properties and reflects the ability to form adducts with nucleophilic sulfhydryl, primary amino, and histydyl groups of proteins, which cause changes in protein structure and function (e.g., 4-hydroxynonenal inhibits glutathione peroxidase) (Mitchell and Petersen 1987). Changes in protein structures and function are additionally enhanced by specific reactions between malondialdehyde or 4-hydroxynonenal and selenocysteine residue of the active center of glutathione peroxidase (Kinter and Roberts 1996), which leads to reduction of its activity (Bosch-Morell 1999). It is believed that glutathione peroxidase is mainly responsible for degradation of the lipid peroxidation products. Independently of reaction with protein moiety, aldehydes generated during lipid peroxidation form couplings with glutathione—main cellular nonenzymatic antioxidant and cosubstrate of glutathione peroxidase. In such a way a decrease in GSH-dependent antioxidant mechanisms of lipid protection leads to enhanced lipid peroxidation. However, a reaction of 4-hydroksynonenal with the sulphydryl group located in the active center of cathepsin B may result in a decrease in cellular activity of this lysosomal protease (O'Neil et al. 1997), observed in this study. The decrease in cathepsin B activity and the increased level of modified structural proteins lead to lower protein degradation in lysosomes and accumulation of oxidatively modified proteins.

Oxidative modifications of phospholipids as well as proteins lead also to disturbances in structure and function of biological membranes, including changes in membrane fluidity and permeability. The decrease in ATP synthesis additionally contributes to membrane destabilization during *F. hepatica* infection (Lenton et al. 1995). It is suggested that lysosomes and cellular membranes are partially disrupted, causing release of lysosomal hydrolases into the cytosol and next into extracellular space (Kalra et al. 1988). Observed in this study the increase in the blood activity of AST and ALT, the markers of liver destruction, confirms belief that *F. hepatica* infection induces oxidative stress, which leads to membrane disturbances and leak of cathepsin B into cytosol and into extracellular space. Cathepsin B released into extracellular space may play a promoting role in carcinogenesis, which is connected with its proteolytic effects on basement membrane and interstitial stroma (Skrzydlewska et al. 2005) and with activation of the urokinase-type proplasminogen, which can subsequently activate the plasmin-metalloproteinase proteolytic pathway (Ikeda et al. 2000). However, unlike other liver flukes, such as *Opisthorchis viverini* or *Clonorchis sinensis*, fasciolosis does not cause carcinogenic action.

It should be emphasized that the ROS influence on the host organism, which is manifested by modification of the host cells components, is probably much different from the ROS influence on parasite cells. *F. hepatica* has been revealed to demonstrate high resistance to ROS and RNS activity (Piedrafita et al. 2000; Sibille et al. 2004), which is mostly associated with a high level of antioxidant, protein ones in particular.

In conclusion, oxidative stress generated in host organism during *F. hepatica* infection leads to oxidative modifications of liver proteins and lipids, which results in disturbances in their functions and cellular metabolism.
